# Prognostic value of plasma level of superoxide dismutase in HBV-related acute-on-chronic liver failure

**DOI:** 10.1186/s12876-022-02371-1

**Published:** 2022-06-25

**Authors:** Naijuan Yao, Yajuan He, Yuchao Wu, Fei Wang, Zhen Tian

**Affiliations:** 1 Department of Infectious Diseases, The First Affiliated Hospital of Xi’an Jiaotong University, Xi’an City, Shaanxi Province China; 2 Department of Ultrasound, The First Affiliated Hospital of Xi’an Jiaotong University, Xi’an City, Shaanxi Province China; 3 Department of Ultrasound and Infectious Diseases, The First Affiliated Hospital of Xi’an Jiaotong University, 277 West Yanta Road, Xi’an City, 710061 Shaanxi Province China

**Keywords:** Acute on chronic liver failure, HBV, Entecavir, Prognosis

## Abstract

**Background:**

Hepatitis B virus-related acute-on-chronic liver failure (HBV-ACLF) is the most prevalent type of ACLF in China. The mortality rate of HBV-ACLF has decreased in recent years due to advances in treatment therapies; however, it is still above 50%. Many cases of HBV-ACLF are caused by HBV reactivation due to discontinuation of nucleoside analog treatment. The present study focused on plasma levels of superoxide dismutase (SOD) in HBV-ACLF patients and investigated whether the plasma level of SOD is a useful biomarker in assessing disease severity and predicting outcomes of HBV-ACLF patients, including patients treated with Entecavir (ETV) and patients who were withdrawn from ETV treatment.

**Methods:**

Plasma samples and clinical data from 200 HBV-ACLF patients and from age- and sex-matched cirrhotic and healthy controls were collected and analyzed. Plasma levels of SOD were measured using an ELISA commercial kit.

**Results:**

Among the HBV-ACLF patients, in the ETV withdrawal group, the mortality rate was higher than in the ETV group (69.95% vs 46.71%, *P* < 0.05). Moreover, HBV-DNA and SOD plasma levels were higher in the ETV withdrawal group than in the ETV group (*Log*_*10*_*(HBV-DNA)*: 6.49 ± 0.24 vs 4.79 ± 0.14, *P* < 0.01; SOD: 463.1 ± 27.61 U/mL vs 397.2 ± 10.97 U/mL, *P* < 0.05). The mortality and liver transplantation rates were significantly higher in HBV-ACLF patients with plasma levels of SOD > 428 U/mL than in patients with plasma SOD levels ≤ 428 U/mL.

**Conclusions:**

Reactivation of HBV and elevated oxidative stress caused by discontinuation of ETV treatment are crucial factors in the pathogenesis of HBV-ACLF. Plasma level of SOD may serve as a useful biomarker in estimating disease severity and predicting outcomes of HBV-ACLF patients who stop ETV treatment.

## Introduction

Acute-on-chronic liver failure (ACLF) is a serious clinical syndrome with liver function deterioration, and high short-time mortality in patients with chronic liver diseases [1]. Hepatitis B virus (HBV) infection is one of the most challenging health problems in the world (especially in Asia), and more than 350 million people are currently chronically infected worldwide [2]. Chronic HBV (CHB) infection causes a spectrum of disease, such as liver cirrhosis, hepatocellular carcinoma (HCC), and liver failure. In most Asian countries, almost 70% of ACLF patients are HBV-infected, while in American and European countries, alcoholic liver disease (ALD) and nonalcoholic fatty liver disease (NAFLD) are the most common underlying liver diseases [3]. In China, HBV-related ACLF (HBV-ACLF) accounts for over 80% of ACLF cases, as almost 7.18% of adults are positive for HBV surface antigen [4].

Nucleoside analogs (NAs) are used to treat CHB infection and effectively inhibit HBV replication by suppressing HBV-polymerase activity. Some recent studies have shown that compared to the non-NA treated group, treatment with NAs, including entecavir (ETV), lamivudine (LVD) and telbivudine (LDT), alleviates severity and reduces mortality in HBV-ACLF patients [5–7]. However, all of these drugs (including ETV, LVD and LDT) only inhibit HBV reverse transcription rather than eliminating the virus. Many cases of HBV-ACLF are caused by HBV reactivation due to withdrawal of NA treatment, as many CHB patients do not receive standard antiviral treatments [8]. Although cessation of NA therapy can cause ACLF and lead to tragic consequences in CHB patients, disease severity and outcome of HBV-ACLF caused by NA withdrawal has not been evaluated.

ACLF usually develops following a precipitating event, including direct liver injuries or extrahepatic insults. Flare-up of CHB infection caused by HBV reactivation due to inappropriate withdrawal of NA treatment, NA resistance, or chemotherapy is the most common acute insult precipitating HBV-ACLF [9]. King’s College Criteria, the SOFA score, and the MELD score are currently widely used in estimating disease severity and outcomes of liver failure patients [10]. A recent study showed that the MELD score can be used to assess disease severity and predict outcomes of HBV-ACLF patients [11]; however, all of the patients enrolled had been treated with NAs (including ETV and LVD) continuously, and it is not clear whether the MELD score serves as a prognostic marker in HBV-ACLF patients when the disease is caused by NA withdrawal.

A recent study showed that excessive inflammation plays an important role during the pathogenesis of many human diseases, including liver failure [12]. Inflammation is thought to play a protective role and to contribute to antimicrobial responses, while excessive inflammation may result in cell injury and cell death. Reactive oxygen species (ROS) have been shown to be crucial for NLRP3 initiation and inflammation activation during ACLF pathogenesis [13]. Superoxide dismutase (SOD) transforms toxic superoxide into hydrogen peroxide and is an important endogenous antioxidant enzyme against intracellular oxidative stress [14]. In our previous study, we found that the plasma SOD level can serve as a valid marker in assessing severity and predicting outcomes of ALF patients based on its simplicity and accuracy [15]. However, it is not clear whether the plasma level of SOD is related to disease severity and outcomes of HBV-ACLF patients, especially patients in whom the disease is caused by ETV withdrawal.

## Materials and methods

### Patients

All of the participants, including HBV-ACLF patients, cirrhotic patients, and healthy individuals, provided written informed consent. This study was approved by the Clinical Research Ethics Committee of the First Affiliated Hospital of Xi’an Jiaotong University. A total of 200 patients diagnosed with HBV-ACLF were enrolled in our study at the First Affiliated Hospital of Xi’an Jiaotong University between January 2013 and September 2017.

These HBV-ACLF patients were divided into three groups based on their usage of ETV: (1) the ETV group: patients who took 0.5 mg ETV daily for more than 1 year (n = 137); (2) the ETV withdrawal group: patients who had been on ETV therapy for at least 1 year and stopped ETV treatment within 6 months of hospital admission (n = 47); and (3) the non-NA group: patients who never took any NAs (n = 16).

### Diagnosis of HBV-ACLF

In total, 245 patients met the diagnostic criteria for ACLF by the Asian Pacific Association for the Study of the Liver (APASL) for CHB infection [16].

The inclusion criteria:

(1) Age: 18–75 years old; (2) plasma levels of bilirubin: ≥ 85 mol/L; (3) coagulation function: international normalized ratio (INR) ≥ 1.5 or prothrombin activity ≤ 40%; (4) encephalopathy and/or clinical ascites within 4 weeks of hospital admission; (5) evidence of ongoing chronic liver diseases.

In total, 45 HBV-ACLF patients were excluded.

The exclusion criteria:

(1) Diagnosis with decompensated liver cirrhosis before ACLF; (2) Portal hypertension with transjugular intrahepatic portosystemic shunt (TIPS); (3) Diagnosed with hepatocellular carcinoma; (4) Other malignancies such as gastric cancer; (5) Pregnancy; (6) Human immunodeficiency virus (HIV) or other hepatotropic viruses than HBV.

The MELD score was calculated with the following standard formula: 11.2*ln (INR) + 9.57*ln (creatinine, mg/dL) + 3.78*ln (bilirubin, mg/dL) + 6.43, with a lower limit of 1 for all of the variables.

As controls, 30 age- and sex-matched healthy individuals and 30 patients with type B hepatitis-related compensate cirrhosis were enrolled.

### Estimation of plasma levels of SOD

Plasma levels of SOD were measured using commercial ELISA kits (*#EIASODC, Thermo Fisher Scientific, Waltham, MA, USA*) according to the manufacturer's protocol. Samples and standards were measured in duplicate, and the sensitivity of the assay was 0.044 U/mL.

Levels of HBV-DNA were measured upon admission in the Department of Clinical Laboratory at the First Affiliated Hospital of Xi’an Jiaotong University.

### Statistical methods

Clinical data are expressed as mean ± standard deviation (mean ± SD). The Chi-squared test or Fisher’s exact test was used to compare categorical variables, and the Wilcoxon rank sum test was used to compare continuous variables. The ROC curve of death or transplantation at 90 days after admission was analyzed by logistic regression. Based on the maximum value for sensitivity and specificity, a cut-off value of continuous variables was determined. The difference between Kaplan–Meier's survival curves at 90 days after admission was compared by log-rank test. SPSS version 16.0 software (*IBM Corporation, Somers, NY, USA*) was used, and differences were of statistical significance when the *P* < 0.05.

## Results

### Baseline characteristics

In total, 200 HBV-ACLF patients, 30 cirrhotic patients, and 30 healthy individuals were included in the present study. Clinical data of all the enrolled participants are shown in Table 1.

**Table 1:** Demographic data and clinical characteristics

Parameter	Healthy individuals	Cirrhotic controls	ACLF-ETV treated	ACLF-ETV withdrawal	ACLF-NAs free
	(n = 30)	(n = 30)	(n = 137)	(n = 47)	(n = 16)
Age (years)	41.13 ± 6.77	44.14 ± 10.65	46.33 ± 13.65	43.70 ± 13.69	46.43 ± 13.93
Sex (M/F)	25/5	25/5	115/23	39/8	12/4
PTA (%)	85.17 ± 12.32	76.15 ± 17.21	35.01 ± 15.40	32.87 ± 15.17	25.14 ± 15.07
FIB (g/L)	2.58 ± 0.61	2.14 ± 0.56	1.51 ± 0.76	1.53 ± 0.76	1.66 ± 0.74
INR	1.16 ± 0.16	1.16 ± 0.14	2.38 ± 1.32	2.87 ± 1.33	2.95 ± 1.36
WBC (10^9^/L)	5.51 ± 1.23	4.25 ± 1.55	6.69 ± 2.51	6.44 ± 2.26	7.08 ± 2.38
PLT (10^9^/L)	201.97 ± 38.25	113.29 ± 74.24	102.16 ± 64.70	83.63 ± 64.79	130.47 ± 65.64
ALT (U/L)	21.32 ± 14.23	33.81 ± 21.39	586.89 ± 744.14	431.73 ± 752.23	938.76 ± 706.13
TBIL (μM)	11.22 ± 3.74	19.26 ± 9.87	328.99 ± 129.40	360.04 ± 130.81	336.34 ± 131.14
CHOL (mM)	3.76 ± 0.57	3.41 ± 0.88	2.13 ± 1.51	1.99 ± 1.51	2.23 ± 1.57
CREA (μM)	50.67 ± 10.35	53.81 ± 11.43	77.88 ± 52.85	72.32 ± 53.15	77.38 ± 54.65
MELD			24.00 ± 6.23	26.00 ± 6.24	25.87 ± 6.19

ALT, alanine aminotransferase; CHOL, cholesterol; CREA, creatinine; FIB, fibrinogen; GLU, glucose; INR, international normalized ratio; Na, sodium ions; PLT, platelet count; PTA, prothrombin activity; TBIL, total bilirubin; WBC, white blood cell count

The most frequent causes of acute hepatic insult were bacterial infection (64.96%) in ETV-treated HBV-ACLF patients and HBV reactivation (100%) in HBV-ACLF patients who withdrew from ETV treatment.

There are many complications in HBV-ACLF such as hepatic encephalopathy (HE), acute kidney injury (AKI), acute variceal bleed, and spontaneous bacterial peritonitis (SBP). HE was the most frequent complication (35.50%) and 24.50% fulfilled the AKI criteria at admission. Baseline clinical data and in-hospital complications are summarized in Table 2.

**Table 2:** Baseline characteristics of ACLF patients

Variables	ACLF	ACLF-ETV	ACLF-ETV	ACLF-NAs
		Treated	Withdrawal	Free
	(n = 200)	(n = 137)	(n = 47)	(n = 16)
*ACLF etiology*				
Acute hepatic insult, n (%)				
Alcoholic hepatitis		38 (27.74%)		4 (25.00%)
HBV reactivation			47 (100%)	
Bacterial infection		89 (64.96%)		9 (56.25%)
Others		10 (7.30%)		3 (18.75%)
*Clinic events, n *(%)				
Ascites	200 (100%)	137 (100%)	47 (100%)	16 (100%)
Jaundice	200 (100%)	137 (100%)	47 (100%)	16 (100%)
AKI	49 (24.50%)	28 (20.44%)	17 (36.17%)	4 (25.00%)
HE	71 (35.50%)	46 (33.58%)	20 (42.55%)	5 (31.25%)
Acute variceal bleed	26 (13.00%)	15 (10.95%)	9 (19.15%)	2 (12.50%)
SBP	15 (7.50%)	9 (6.57%)	5 (10.64%)	1 (6.25%)
*Organ failure*				
Kidney, n (%)	19 (9.50%)	12 (8.76%)	5 (10.64%)	2 (12.50%)
Cerebral, n (%)	33 (16.50%)	20 (14.60%)	10 (21.28%)	3 (18.75%)
Coagulation, n (%)	70 (35.00%)	46 (33.58%)	19 (40.43%)	5 (31.25%)
Circulation, n (%)	25 (12.50%)	16 (11.68%)	7 (14.89%)	2 (12.50%)
Lung, n (%)	60 (30.00%)	38 (27.74%)	17 (36.17%)	5 (31.25%)

### Withdrawal of ETV led to elevated mortality rates in HBV-ACLF patients

**Fig. 1:**
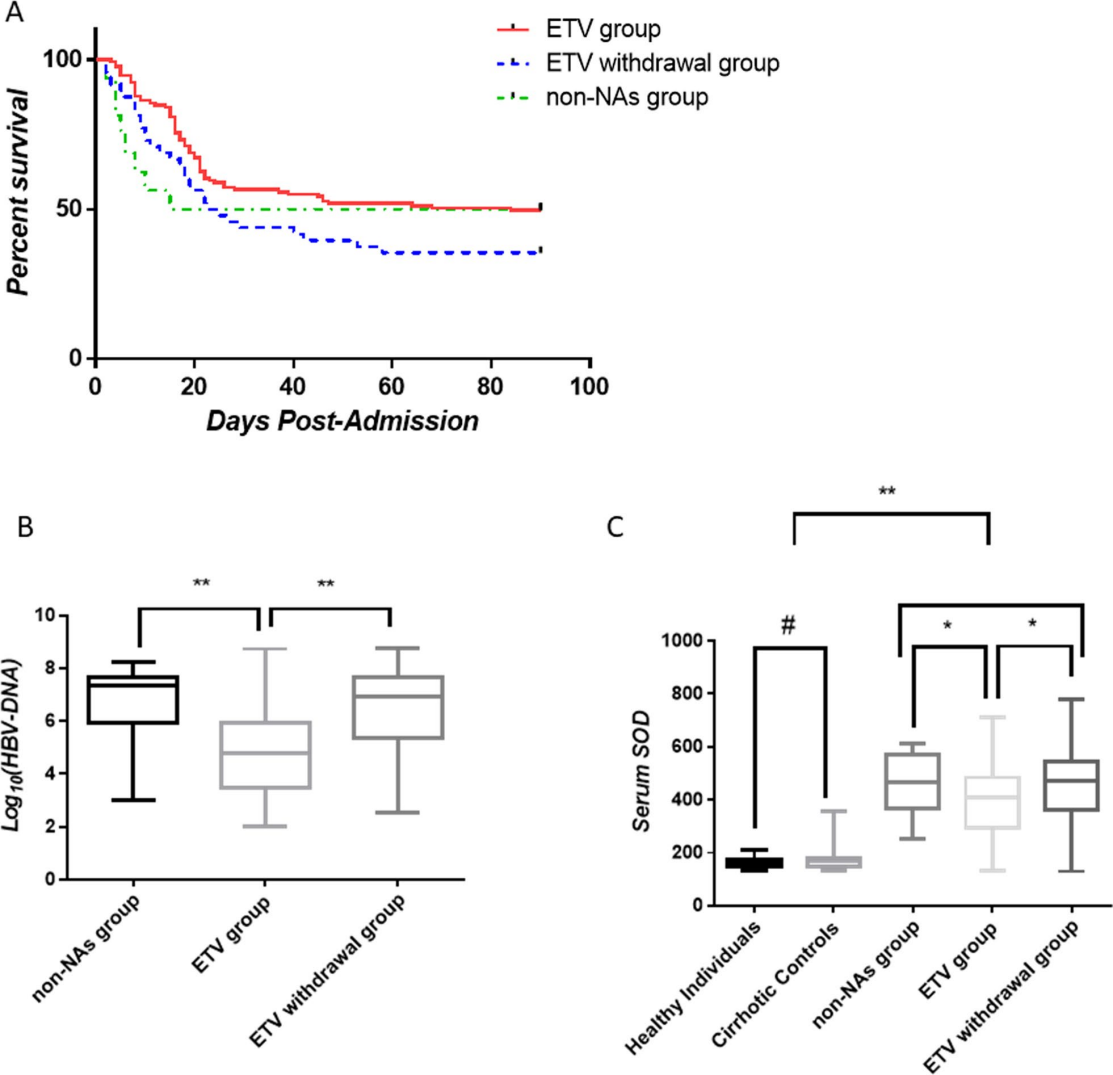
Clinical characteristics of controls and ACLF patients. **a** Kaplan–Meier survival analysis in ACLF patients; **b** Serum HBV-DNA levels in ACLF patients; **c** Serum SOD levels in controls and ACLF patients. *P < 0.05, **P < 0.01, ^#^*P* > 0.05

The mortality rate of HBV-ACLF patients at 90 days was 46.71% in the ETV group, and 65.95% in the ETV withdrawal group. Statistically significant differences were found between the survival curves of patients in the ETV and ETV withdrawal groups. Among the 16 HBV-ACLF patients who did not take any NAs or interferon, 8 patients died at 90 days (Fig. 1A).

### Withdrawal of ETV led to elevated plasma HBV-DNA levels

Withdrawal of NAs leads to reactivation of HBV. Therefore, we compared *Log*_*10*_*(HBV-DNA)* values among different groups. A significant difference was found between patients in the ETV and ETV withdrawal groups (4.79 ± 0.14 vs 6.49 ± 0.24, *P* < 0.01). Moreover, a significant difference was found between the ETV and non-NA groups (4.79 ± 0.14 vs 6.83 ± 0.34, *P* < 0.01) (Fig. 1B).

In the present study we found that plasma levels of SOD were significantly higher in patients with HBV-ACLF compared to healthy individuals (412.1 ± 9.90 U/mL vs 164.2 ± 3.82 U/mL, *P* < 0.01) and to cirrhotic controls (412.1 ± 9.90 vs 176.7 ± 8.08, *P* < 0.01). We also found that plasma SOD levels were higher in the non-NA group compared to the ETV group (446.0 ± 30.69 U/mL vs 397.2 ± 10.97 U/mL, *P* < 0.05), and it was higher in the ETV withdrawal group compared to the ETV group (463.1 ± 27.61 U/mL vs 397.2 ± 10.97 U/mL, *P* < 0.05). No significant differences were found between ETV withdrawal and non-NA groups (446.0 ± 30.69 U/mL vs 463.1 ± 27.61 U/mL, *P* = 0.70) (Fig. 1C).

### Plasma SOD level was an independent risk factor for HBV-ACLF mortality

Here, we identified the potential risk factors for HBV-ACLF patients using univariate and multivariate Cox regression analyses. As shown in Table 3, INR (HR = 2.703, 95% CI 1.784–4.097, *P* < 0.01), CHOL (HR = 0.704, 95% CI 0.497–0.997, *P* < 0.05), CREA (HR = 1.011, 95% CI 1.001–1.021, *P* < 0.05), MELD score (HR = 1.364, 95% CI 1.228–1.475, *P* < 0.01), plasma SOD level (HR = 1.011, 95% CI 1.008–1.015, *P* < 0.01), and Log_10_(HBV-DNA) (HR = 1.271, 95% CI 1.033–1.433, *P* < 0.01) were significantly associated with mortality in ACLF patients. We next performed a forward multivariate analysis including CHOL, MELD score, plasma SOD and Log_10_(HBV-DNA). The results revealed that MELD score (HR = 1.314, 95% CI 1.185–1.457, *P* < 0.01), and plasma SOD level (HR = 1.011, 95% CI 1.007–1.014, *P* < 0.01) were independent risk factors for mortality in ACLF patients.

**Table 3:** Uni-and multivariate logistic analysis of prognosis factors associated with survival in patients with HBV-ACLF

	Univariate			Multivariate		
	HR	95% CI	*P*	HR	95% CI	*P*
Age (year)	1.055	1.028–1.082	0.124			
Sex (M/F)	0.873	0.420–1.815	0.717			
PT (%)	1.069	1.030–1.109	0.422			
Fb (g/L)	0.811	0.540–1.218	0.312			
INR	2.703	1.784–4.097	< 0.01			
WBC (1 × 10^9^/L)	1.000	0.998–1.001	0.518			
PLT (1 × 10^9^/L)	0.992	0.986–0.998	0.079			
ALT (U/L)	1.000	1.000–1.000	0.456			
TBIL (μM)	1.006	1.004–1.009	0.133			
CHOL (mM)	0.704	0.497–0.997	0.048	1.229	0.711–2.127	0.460
CREA (μM)	1.011	1.001–1.021	0.033			
MELD	1.364	1.228–1.475	< 0.01	1.314	1.185–1.457	< 0.01
Plasma SOD (U/mL)	1.011	1.008–1.015	< 0.01	1.011	1.007–1.014	< 0.01
HBV-DNA (*Log*_*10*_)	1.217	1.033–1.433	0.019	1.133	0.910–1.410	0.265

### Plasma SOD level and MELD score were associated with mortality or liver transplantation in HBV-ACLF patients

**Fig. 2:**
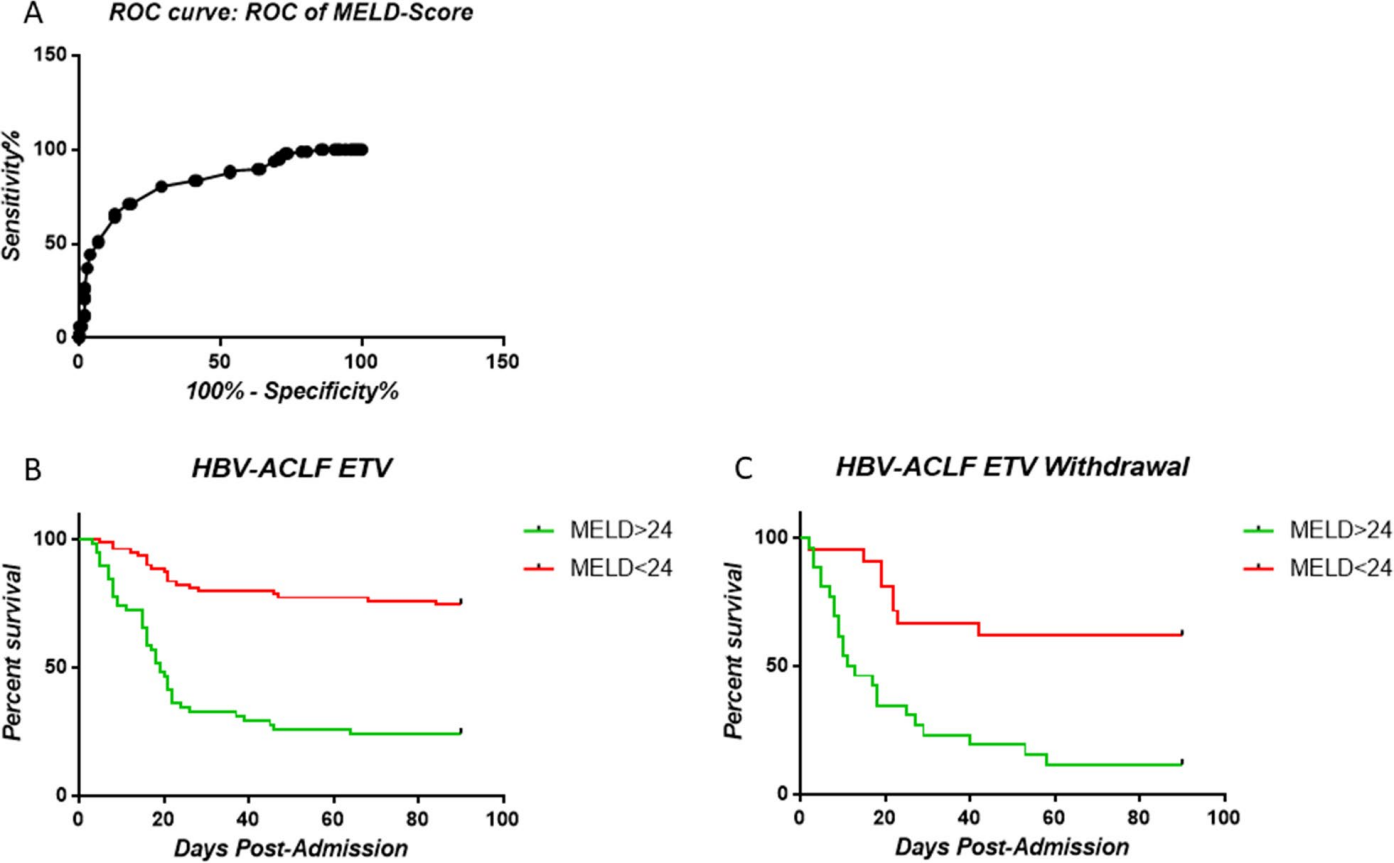
Kaplan–Meier survival analysis according to the MELD score at admission. **a** ROC curve of the MELD score; **b** Survival of ACLF patients in the ETV group according to the MELD score; **c** Survival of ACLF patients in the ETV withdrawal group according to the MELD score

In the current cohort, 9 patients underwent liver transplantation, and another 94 died without transplantation. The MELD score is one of the most useful scoring systems in assessing severity and disease outcome of liver failure patients. We previously found that ALF patients with MELD scores above 25 showed significantly higher mortality and liver transplantation rates. Here, we found that HBV-ACLF patients with l MELD scores above 24 showed significantly higher mortality and liver transplantation rates within 90 days by using ROC methodology. (Fig. 2A–C).

**Fig. 3:**
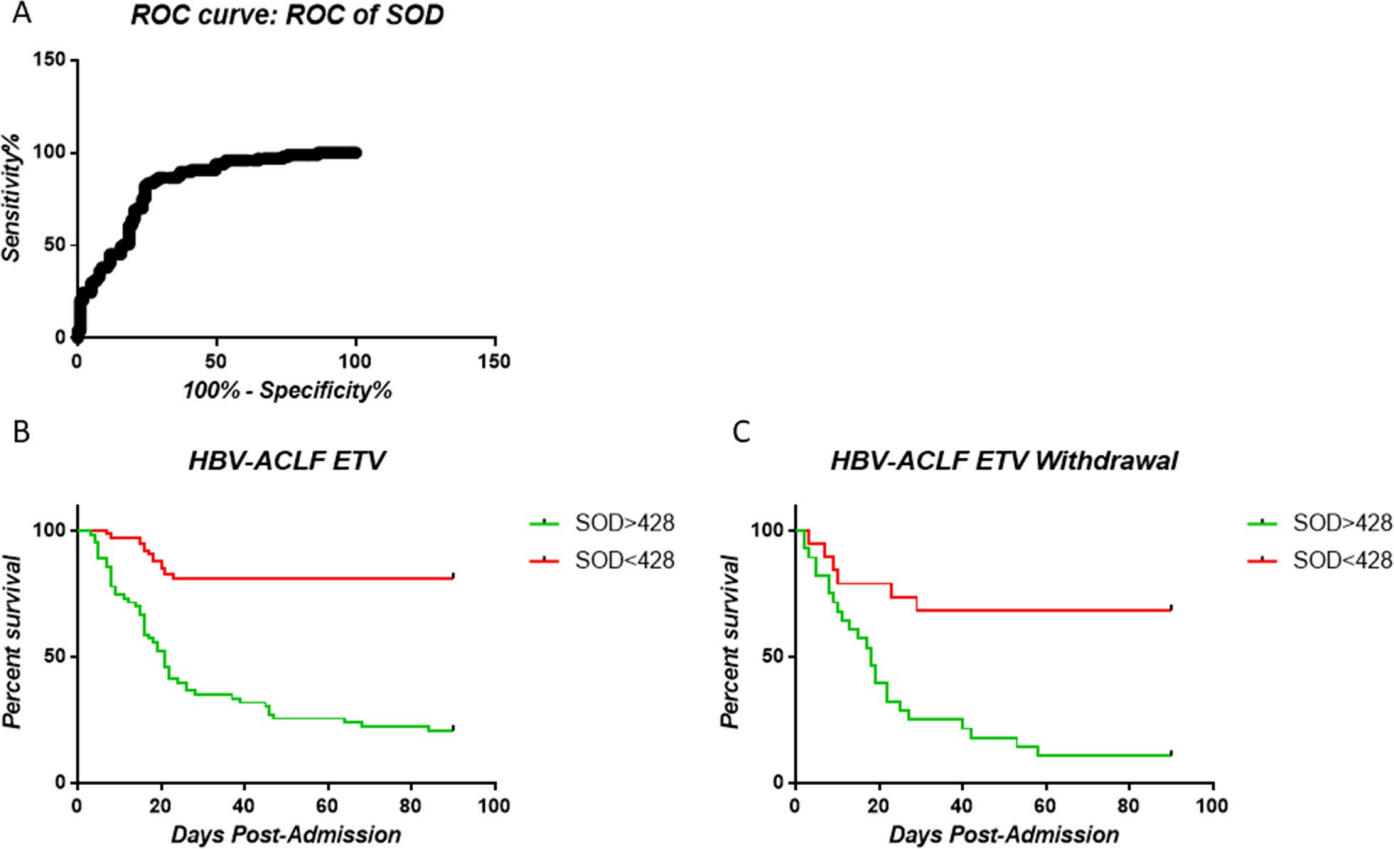
Kaplan–Meier survival analysis according to SOD level at admission. **a** ROC curve of plasma SOD; **b** Survival of ACLF patients in the ETV group according to plasma SOD level; **c** Survival of ACLF patients in the ETV withdrawal group according to plasma SOD level

We also found that HBV-ACLF patients with plasma levels of SOD above 428 U/mL showed significantly higher mortality and liver transplantation rates within 90 days by using ROC methodology. (Fig. 3A–C).

We next compared the AUC values of plasma SOD level, the MELD score and CRE. SOD was found to be a better marker in predicting disease outcome of HBV-ACLF patients than MELD and CRE with an AUC of 0.8411 (Table 4).

**Table 4:** Predictive values of laboratory parameters for prognosis in patients with ACLF

Parameter	AUC	*P* value	Cut-off	Sensitivity	Specificity	95% CI for AUC	
						Lower	Upper
CRE	0.5817	0.046	63.00	55.67	60.19	50.26	66.07
MELD	0.7927	< 0.01	24.00	71.13	81.55	72.70	83.51
SOD	0.8411	< 0.01	428.00	81.44	79.73	66.29	88.64

Next, the 200 HBV-ACLF patients were divided into the low SOD group (SOD < 428 U/mL) and the high SOD group (SOD > 428 U/mL) according to plasma SOD level at hospital admission. The differences in clinical and laboratory characteristics between the two groups are listed in Table 5. The MELD score in the high SOD group was higher than in the low SOD group. Furthermore, patients with higher values of SOD had higher INR, WBC, TBIL, and HBV-DNA levels and higher mortality. The PT, Fb, PLT, ALT, CHOL, and CREA levels were not significantly different between the two groups.

**Table 5:** Clinical and laboratory characteristics among patients with different SOD values at hospital admission

	Low group	High group	*P*
	(SOD < 428 U/mL, n = 104)	(SOD > 428 U/mL, n = 96)	
Age (year)	44.40 ± 1.27	47.15 ± 1.33	0.138
PT (%)	25.09 ± 1.11	27.75 ± 1.13	0.095
Fb (g/L)	1.55 ± 0.07	1.50 ± 0.07	0.574
INR	2.33 ± 0.13	2.77 ± 0.13	< 0.01
WBC (1 × 10^9^/L)	6.39 ± 0.30	7.54 ± 0.50	0.048
PLT (1 × 10^9^/L)	105.19 ± 5.46	94.94 ± 5.22	0.177
Neutrophil count (1 × 10^9^/L)	8.48 ± 0.96	9.24 ± 0.89	0.571
ALT (U/L)	573.10 ± 76.44	584.54 ± 92.86	0.924
TBIL (μM)	293.67 ± 13.21	383.69 ± 12.19	< 0.01
CHOL (mM)	2.17 ± 0.08	2.03 ± 0.08	0.253
CREA (μM)	73.61 ± 2.79	79.70 ± 3.89	0.199
MELD	22.72 ± 0.57	26.60 ± 0.54	< 0.01
HBV-DNA (*Log*_*10*_)	5.07 ± 0.17	5.67 ± 0.19	0.017

## Discussion

ACLF is a serious clinical syndrome with liver function deterioration, and is associated with a high short-time mortality rate. The mortality rate of HBV-ACLF has decreased in recent years due to advances in treatment therapies. In the present study, we found a 54.85% mortality rate in all of the HBV-ACLF patients. Currently, CHB infection remains an important infectious disease in the Asian-Pacific region, especially in China, and several NAs are available for the treatment of HBV replication in CHB patients. In China, a shortage of medical assistance, a lack of medical knowledge, or fear of social prejudices leads to discontinuation of NA treatment, which finally contributes to HBV rebound and liver failure [4]. We found that HBV-ACLF patients in the ETV withdrawal and non-NA groups had significantly higher mortality rates compared to ETV treated patients.

Treatment with ETV has been shown to be effective in alleviating disease severity and increasing survival rates in HBV-ACLF patients [5]. Antiviral therapy effectively inhibits active HBV replication and improves hepatic function. In addition, many cases of HBV-ACLF are caused by HBV reactivation due to withdrawal of NA treatment [6, 17]. We found an increased level of plasma HBV-DNA in the ETV withdrawal group in our present study; ETV withdrawal leads to HBV reactivation and hence increased HBV-DNA levels, initiating HBV-ACLF.

Oxidative stress is common in various types of liver injury, and plays a critical role in the mechanism of liver failure [18, 19]. Overactivation of oxidative stress could lead to hepatocyte injury and/or death, and the injured and/or dead hepatocytes then cause oxidative stress, which contributes to further hepatocyte loss [18]. Also, oxidative stress has been shown to be important in activating the initial steps of various disorders, including hepatic failure. As adaptive responses to overactivation of oxidative stress in HBV-ACLF, elevated plasma levels of SOD have been found in HBV-ACLF patients. ACLF patients with plasma levels of SOD above 428 U/mL showed significantly higher mortality and liver transplantation rates within 90 days. Moreover, we found significantly higher plasma levels of SOD in the ETV withdrawal and non-NA groups. HBV infection triggers oxidative stress on hepatocytes, which then cascades to a systematic response [20, 21]. Virus infection is associated with oxidative stress, as previous studies showed that cultured cells infected with HIV, influenza virus or HCV had increased ROS levels [22]. In the pathogenesis of hepatitis B, ongoing extensive oxidative stress occurs. Several studies have linked HBV to oxidative stress levels [23, 24]. It is believed that HBV generates oxidative stress via altering mitochondrial function and modulating host gene expression [24–27].

As we previously mentioned, the MELD score, which focuses only on damaged liver function, has been widely applied in assessing disease severity and outcomes of HBV-ACLF patients. The MELD score is determined based on three components: bilirubin, which is related to liver function, creatinine, which is related to renal function, and INR, which is related to coagulation function. Therefore, the predictive value of the MELD score is limited by laboratory variability [11]. In the present study, we confirmed the predictive value of the MELD score for the prognosis of HBV-ACLF patients. We found that HBV-ACLF patients with MELD scores above 24 showed significantly higher mortality and liver transplantation rates within 90 days. We also analyzed the prognostic value of plasma level of SOD and the MELD score in HBV-ACLF patients, and we found that SOD had higher discriminative power and higher AUC value than the MELD score in assessing disease severity and predicting outcome. Since oxidative stress plays a central role during HBV-ACLF, the increased oxidative stress due to HBV reactivation in the ETV withdrawal and non-NA groups leads to much higher mortality rates compared to ETV treated HBV-ACLF patients.

## Conclusion

The present study revealed increased mortality rates and HBV-DNA levels in ETV withdrawal HBV-ACLF patients. We also found increased levels of SOD in ETV withdrawal patients. HBV initiated the hepatic failure process and generated oxidative stress, which plays a central role in hepatic failure pathogenesis, resulting in high mortality rates. Early SOD rather than HBV-DNA levels served as a predictor of disease severity and outcomes of HBV-ACLF patients. In addition, our study provides insight into the underlying pathogenic mechanisms, supporting the development of strategies to modulate HBV or oxidative stress and the identification of potential targets for treating HBV-ALCF patients.

## Data Availability

The datasets used and/or analyzed during the current study are available from the corresponding author on reasonable request.

## Abbreviations

ACLF: Acute-on-chronic liver failure
HBV: Hepatitis B virus
CHB: Chronic hepatitis B
HCC: Hepatocellular carcinoma
ALD: Alcoholic liver disease
NAFLD: Nonalcoholic fatty liver disease
HBV-ACLF: HBV-related ACLF
NAs: Nucleoside analogues
ETV: Entecavir
ROS: Reactive oxygen species
SOD: Superoxide dismutase
TIPS: Transjugular intrahepatic portosystemic shunt
HIV: Human Immunodeficiency Virus
HE: Hepatic encephalopathy
AKI: Acute kidney injury
SBP: Spontaneous bacterial peritonitis

## References

[1] Blasco-Algora S, Masegosa-Ataz J, Gutiérrez-García ML, Alonso-López S, Fernández-Rodríguez CM (2015). Acute-on-chronic liver failure: pathogenesis, prognostic factors and management. World J Gastroenterol.

[2] Sundaram V, Kowdley K (2015). Management of chronic hepatitis B infection. BMJ.

[3] Zhao RH, Shi Y, Zhao H, Wu W, Sheng JF (2018). Acute-on-chronic liver failure in chronic hepatitis B: an update. Expert Rev Gastroenterol Hepatol.

[4] Xie GJ, Zhang HY, Chen Q, Liu HM, You JP, Yang S, Mao Q, Zhang XQ (2016). Changing etiologies and outcome of liver failure in Southwest China. Virol J.

[5] Chen T, He Y, Liu X, Yan Z, Wang K, Liu H, Zhang S, Zhao Y (2012). Nucleoside analogues improve the short-term and long-term prognosis of patients with hepatitis B virus-related acute-on-chronic liver failure. Clin Exp Med.

[6] He D, Guo S, Chen W (2013). Long-term outcomes after nucleos(t)ide analogues discontinuation in chronic hepatitis B patients with HBeAg-negative. BMC Infect Dis.

[7] Wang J, Ma K, Han M (2014). Nucleoside analogs prevent disease progression in HBV-related acute-on-chronic liver failure: validation of the TPPM model. Hepatol Int.

[8] Lin PC, Poh SB, Lee MY, Hsiao LT, Chen PM, Chiou TJ (2005). Fatal fulminant hepatitis B after withdrawal of prophylactic lamivudine in hematopoietic stem cell transplantation patients. Int J Hematol.

[9] Bernal W, Jalan R, Quaglia A, Simpson K, Wendon J, Burroughs A (2015). Acute-on-chronic liver failure. Lancet.

[10] Wu FL, Shi KQ, Chen YP, Braddock M, Zou H, Zheng MH (2014). Scoring systems predict the prognosis of acute-on-chronic hepatitis B liver failure: an evidence-based review. Expert Rev Gastroenterol Hepatol.

[11] Luo Y, Xu Y, Li M, Xie Y, Gong G (2016). A new multiparameter integrated MELD model for prognosis of HBV-related acute-on-chronic liver failure. Medicine (Baltimore).

[12] Mehta G, Mookerjee RP, Sharma V, Jalan R (2015). Systemic inflammation is associated with increased intrahepatic resistance and mortality in alcohol-related acute-on-chronic liver failure. Liver Int.

[13] Sho T, Xu J (2019). Role and mechanism of ROS scavengers in alleviating NLRP3-mediated inflammation. Biotechnol Appl Biochem.

[14] Lu X, Wang C, Liu B (2015). The role of Cu/Zn-SOD and Mn-SOD in the immune response to oxidative stress and pathogen challenge in the clam Meretrix meretrix. Fish Shellfish Immunol.

[15] Tian Z, Chen Y, Yao N (2018). Role of mitophagy regulation by ROS in hepatic stellate cells during acute liver failure. Am J Physiol Gastrointest Liver Physiol.

[16] Sarin SK, Kedarisetty CK, Abbas Z (2014). Acute-on-chronic liver failure: consensus recommendations of the Asian Pacific Association for the Study of the Liver (APASL) 2014. Hepatol Int.

[17] Rinker F, Zimmer CL, HönerZuSiederdissen C, Manns MP, Kraft A, Wedemeyer H, Björkström NK, Cornberg M (2018). Hepatitis B virus-specific T cell responses after stopping nucleos(t)ide analogue therapy in HBeAg-negative chronic hepatitis B. J Hepatol.

[18] Li T, Meng QH, Zou ZQ (2011). Correlation between promoter methylation of glutathione-S-tranferase P1 and oxidative stress in acute-on-chronic hepatitis B liver failure. J Viral Hepat.

[19] Borrelli A, Bonelli P, Tuccillo FM, Goldfine ID, Evans JL, Buonaguro FM, Mancini A (2018). Role of gut microbiota and oxidative stress in the progression of non-alcoholic fatty liver disease to hepatocarcinoma: current and innovative therapeutic approaches. Redox Biol.

[20] Alavian SM, Showraki A (2016). Hepatitis B and its relationship with oxidative stress. Hepat Mon.

[21] Zou LY, Zheng BY, Fang XF, Li D, Huang YH, Chen ZX, Zhou LY, Wang XZ (2015). HBx co-localizes with COXIII in HL-7702 cells to upregulate mitochondrial function and ROS generation. Oncol Rep.

[22] Lin W, Wu G, Li S (2011). HIV and HCV cooperatively promote hepatic fibrogenesis via induction of reactive oxygen species and NFkappaB. J Biol Chem.

[23] Jabeen K, Malik U, Mansoor S, Shahzad S, Zahid S, Javed A (2021). Effect of oxidative stress and calcium deregulation on FAM26F (CALHM6) expression during hepatitis B virus infection. BMC Infect Dis.

[24] Ren JH, Chen X, Zhou L (2016). Protective role of Sirtuin3 (SIRT3) in oxidative stress mediated by hepatitis B virus X protein expression. PLoS ONE.

[25] Mansouri A, Gattolliat CH, Asselah T (2018). Mitochondrial dysfunction and signaling in chronic liver diseases. Gastroenterology.

[26] Gao WY, Li D, Cai DE, Huang XY, Zheng BY, Huang YH, Chen ZX, Wang XZ (2017). Hepatitis B virus X protein sensitizes HL-7702 cells to oxidative stress-induced apoptosis through modulation of the mitochondrial permeability transition pore. Oncol Rep.

[27] Severi T, Ying C, Vermeesch JR (2006). Hepatitis B virus replication causes oxidative stress in HepAD38 liver cells. Mol Cell Biochem.

